# Crowdsourced surveillance for neglected tropical diseases, Nigeria

**DOI:** 10.2471/BLT.24.292448

**Published:** 2025-09-03

**Authors:** Uchechukwu Madukaku Chukwuocha, Ayoola Oluwaseun Bosede, Christopher Sule Oyamienlen, Joshua Chisom Ogboeze, David Chinecherem Innocent, Chidera Chisom Obasi, Akajiaku Chukwunyere Chukwuocha, Obiageli Nebe, Sammy Olufemi Sam-Wobo, Chinyere Nneka Ukaga, Ikechukwu Nosike Dozie, Bertram Ekejiuba Nwoke

**Affiliations:** a Innovations and Technologies for Disease Control Research Group, Department of Public Health, Federal University of Technology, PMB 1526 Owerri, Nigeria.; b Department of Surveying and Geoinformatics, Federal University of Technology, Owerri, Nigeria.; c NTDs Elimination Programme, Federal Ministry of Health and Social Welfare, Abuja, Nigeria.; d Department of Animal and Environmental Biology, Federal University of Agriculture, Abeokuta, Nigeria.; e Department of Animal and Environmental Biology, Imo State University, Owerri, Nigeria.; f Department of Microbiology, Kingsley Ozumba Mbadiwe University, Ideato, Nigeria.

## Abstract

**Objective:**

To validate a crowdsourced, image-based morbidity hotspot method for surveillance of neglected tropical diseases.

**Methods:**

We conducted our crowdsourced surveillance pilot implementation study between November 2022 and October 2024 in 45 communities across three Nigerian states, covering a population of 477 138 people. Three additional states, where the project was not implemented but surveillance data obtained, served as control. Residents self-reported suspected symptoms by using smartphones to capture and transmit images of skin and eye manifestations via digital communication platforms. An expert panel then examined the images to confirm signs of neglected tropical diseases. We used frequency and percentages to present data; we also compared incidence data from both pilot and control locations.

**Findings:**

In total, 512 subjects submitted images, either themselves or via a community focal point. Their mean age was 53  years (standard deviation: 20.7). Forty-six percent (234/512) were women and 55% (281/512) were farmers. Notably, 43% (218/512) had experienced symptoms of neglected tropical diseases for 1–5 years before our study and 85% (437/512) had not received any intervention. Of all photos submitted, 75% (386/512) showed signs of neglected tropical diseases. In Ondo state crowdsourced surveillance led to an average of 54.3 monthly reports, versus traditional surveillance which averaged 6.8 (*P* < 0.01). Cost analysis showed that crowdsourced surveillance cost 72.4 United States dollars per case detected.

**Conclusion:**

Our surveillance method outperformed traditional surveillance, showing its promise for enhancing neglected tropical disease surveillance. The method’s ability to detect emerging conditions and support post-elimination surveillance reinforces its value.

## Résumé

**Objectif:**

Valider une méthode participative de surveillance des foyers de morbidité à partir d’images pour les maladies tropicales négligées.

**Méthodes:**

Nous avons mené notre étude pilote de mise en œuvre de la surveillance participative entre novembre 2022 et octobre 2024 dans 45 groupes de population répartis dans trois États nigérians, couvrant un total de 477 138 personnes. Trois autres États, où le projet n’a pas été mis en œuvre mais où des données de surveillance ont été obtenues, ont servi de groupe témoin. Les résidents ont eux-mêmes signalé les symptômes suspects à l’aide de smartphones pour capturer et transmettre des images des manifestations cutanées et oculaires sur des plateformes de communication numériques. Un groupe d’experts a ensuite examiné ces images pour confirmer les signes de maladies tropicales négligées. Nous avons utilisé la fréquence et des pourcentages pour présenter les données. Nous avons également comparé les données d’incidence des sites pilotes et des sites témoins.

**Résultats:**

En tout, 512 personnes ont soumis des images, soit elles-mêmes, soit par l’intermédiaire d’un point de coordination communautaire. Leur âge moyen était de 53 ans (écart type: 20,7). 46% (234/512) d’entre elles étaient des femmes et 55% (281/512) étaient des agriculteurs. Il est à noter que 43% (218/512) avaient présenté des symptômes de maladies tropicales négligées pendant 1 à 5 ans avant notre étude et que 85% (437/512) n’avaient bénéficié d’aucune intervention. Parmi toutes les photos soumises, 75% (386/512) montraient des signes de maladies tropicales négligées. Dans l’État d’Ondo, la surveillance participative a donné lieu à une moyenne de 54,3 rapports mensuels, contre 6,8 pour la surveillance traditionnelle (P < 0,01). L’analyse des coûts a montré que la surveillance participative coûtait 72,4 dollars américains par cas détecté.

**Conclusion:**

Notre méthode de surveillance a surpassé la surveillance traditionnelle, démontrant son potentiel pour améliorer la surveillance des maladies tropicales négligées. La capacité de cette méthode à détecter les affections émergentes et à soutenir la surveillance à l’élimination des affections renforce sa valeur.

## Resumen

**Objetivo:**

Validar un método de detección de focos de morbilidad basado en imágenes obtenidas por colaboración abierta para la vigilancia de las enfermedades tropicales desatendidas.

**Métodos:**

Se llevó a cabo un estudio piloto de implementación de vigilancia mediante colaboración abierta entre noviembre de 2022 y octubre de 2024 en 45 comunidades de tres estados de Nigeria, que abarcó una población de 477 138 personas. Otros tres estados, donde el proyecto no se implementó pero de los que se obtuvieron datos de vigilancia, sirvieron como control. Los residentes autoinformaron síntomas sospechosos utilizando teléfonos inteligentes para capturar y transmitir imágenes de manifestaciones cutáneas y oculares a través de plataformas de comunicación digital. Un panel de expertos examinó las imágenes para confirmar signos de enfermedades tropicales desatendidas. Los datos se presentaron en frecuencias y porcentajes; además, se compararon los datos de incidencia de las zonas piloto y de control.

**Resultados:**

En total, 512 personas remitieron imágenes, ya sea directamente o a través de un punto focal comunitario. La media de edad fue de 53 años (desviación estándar: 20,7). El 46% (234/512) eran mujeres y el 55% (281/512) agricultores. Cabe destacar que el 43% (218/512) había experimentado síntomas de enfermedades tropicales desatendidas durante entre 1 y 5 años antes del estudio y el 85% (437/512) no había recibido ninguna intervención. Del total de fotografías remitidas, el 75% (386/512) mostraban signos de enfermedades tropicales desatendidas. En el estado de Ondo, la vigilancia mediante colaboración abierta generó un promedio de 54,3 notificaciones mensuales, frente a la vigilancia tradicional que promedió 6,8 (*p* < 0,01). El análisis de costos mostró que la vigilancia mediante colaboración abierta tuvo un costo de 72,4 dólares estadounidenses por caso detectado.

**Conclusión:**

El método de vigilancia superó a la vigilancia tradicional, lo que demuestra su potencial para reforzar la vigilancia de las enfermedades tropicales desatendidas. Su capacidad para detectar afecciones emergentes y respaldar la vigilancia posterior a la eliminación refuerza su valor.

## ملخص

الغرض التحقق من صحة طريقة التعهيد الجماعي لرصد معدل الإصابة بالأمراض الاستوائية المهملة، باستخدام الصور.

الطريقة قمنا بإجراء دراسة تنفيذية تجريبية للرصد بالاعتماد على التعهيد الجماعي، بين نوفمبر/تشرين ثاني 2022 وأكتوبر/تشرين أول 2024، في 45 مجتمعًا محليًا موزعًا على ثلاث ولايات نيجيرية، تغطي تعدادًا سكانيًا يبلغ 477138 نسمة. وتم استخدام ثلاث ولايات إضافية لم يُنفذ فيها المشروع، كعامل للتحكم، ولكن تم الحصول على بيانات للرصد فيها. أبلغ المقيمون عن الأعراض المشتبه بها لديهم، باستخدام هواتفهم الذكية لالتقاط صور لحالة الجلد والعين وإرسالها عبر منصات التواصل الرقمية. ثم قامت لجنة من الخبراء بفحص الصور لتأكيد علامات تشير إلى الأمراض الاستوائية المهملة. كما استخدمنا الترددات والنسب المئوية لعرض البيانات؛ وقمنا بمقارنة بيانات الإصابة من كل من مواقع الدراسة التجريبية ومواقع التحكم.

النتائج في المجمل، قدّم 512 مشاركًا صورًا، إما بأنفسهم أو عبر جهة اتصال مجتمعية. وكان متوسط أعمارهم 53 عامًا (الانحراف المعياري: 20.7). ستة وأربعون بالمائة (234/512) كانوا من النساء، و%55 (281/512) كانوا من المزارعين. والجدير بالملاحظة أن %43 (218/512) قد عانوا من أعراض أمراض المناطق الاستوائية المهملة لمدة 1 إلى 5 سنوات قبل دراستنا، ولم يتلق %85 (437/512) أي تدخل. من بين جميع الصور المقدمة، أظهرت %75 (386/512) علامات على أمراض المناطق الاستوائية المهملة. في ولاية أوندو، أدى رصد التعهيد الجماعي إلى متوسط 54.3 تقريرًا شهريًا، في مقابل تقارير الرصد التقليدي التي بلغ متوسطها 6.8 (نسبة الاحتمال أقل من 0.01). أظهر تحليل التكلفة أن تكلفة رصد التعهيد الجماعي هي 72.4 دولارًا أمريكيًا لكل حالة يتم اكتشافها.

الاستنتاج تفوقت طريقة الرصد لدينا على الرصد التقليدي، مما يدل على قدرتها على تعزيز رصد أمراض المناطق الاستوائية المهملة. إن قدرة الطريقة على اكتشاف الحالات المستجدة، ودعم الرصد بعد القضاء عليها، تعزز من قيمتها.

## 摘要

**目的:**

旨在验证适用于监测被忽视的热带病的一种基于众包图像的疾病高发区识别方法。

**方法:**

我们于 2022 年 11 月至 2024 年 10 月期间在尼日利亚三个州的 45 个社区开展了一项众包监测试点实施研究，该项研究总共将 477,138 人纳入了监测范畴。我们将未实施上述项目但仍收集了其相关监测数据的另外三个州作为该项研究的对照组。居民使用智能手机采集皮肤和眼部表现相关图像，并通过数字通信平台传输此类图像，以自行报告其疑似症状。然后，一个专家小组审查了此类图像以确认被忽视的热带病的体征。我们采用了频率和百分比来呈现数据，我们还对试点地和对照地的发病率数据进行了对比。

**结果:**

总共有 512 位研究对象自行或通过社区联络点代为提交了图像。他们的平均年龄是 53 岁（标准偏差：20.7）。其中 46% (234/512) 为女性，55% (281/512) 为农民。值得注意的是，在我们开展该项研究之前，其中 43% (218/512) 已出现了被忽视的热带病症状，时间长达 1-5 年，且 85% (437/512) 未曾接受过任何干预治疗。在提交的所有照片中，75% (386/512) 的研究对象出现了被忽视的热带病体征。通过在尼日利亚翁多州开展的众包监测项目，平均每月收到了 54.3 份报告，而通过传统监测项目，平均每月只收到了 6.8 份报告 (*P* < 0.01)。成本分析结果显示，每检测出一例病例，所需众包监测成本为 72.4 美元。

**结论:**

我们的监测方法优于传统监测方法，这表明前者在加强对被忽视的热带病监测方面的前景更好。该方法能够检测出新出现的病症并支持病症消除后监测，这进一步提升了其价值。

## Резюме

**Цель:**

Провести валидацию метода выявления очагов для оценки заболеваемости по изображениям в ходе надзора за забытыми тропическими болезнями с привлечением технологии краудсорсинга.

**Методы:**

Авторы провели пробное имплементационное исследование надзора с применением технологии краудсорсинга в период между ноябрем 2022 года и октябрем 2024 года в 45 общинах в различных штатах Нигерии; охват населения составил 477 138 человек. В качестве контроля служили три дополнительных штата, в которых проект не реализовывался, но данные надзора были получены. Жители самостоятельно сообщали о наличии подозрительных симптомов и использовали смартфоны, чтобы сфотографировать и передать изображения кожи и глаз посредством цифровых коммуникационных платформ. Затем группа специалистов оценивала изображения, чтобы подтвердить наличие признаков забытых тропических заболеваний. Авторы представили данные с распределением по частоте и процентам. Также сравнивались данные по частоте заболеваемости в группе исследования и контрольной группе.

**Результаты:**

В общей сложности были получены изображения от 512 лиц, которые отправили их самостоятельно или через контактное лицо в общине. Средний возраст составлял 53 года (стандартное отклонение: 20,7). Сорок шесть процентов (234/512) участников составляли женщины, а 55% (281/512) были фермерами. Примечательно, что 43% (218/512) имели симптомы забытых тропических болезней на протяжении 1–5 лет до начала исследования и 85% (437/512) не получали никаких вмешательств. Среди всех представленных снимков на 75% (386/512) наблюдались признаки забытых тропических болезней. В штате Ондо краудсорсинговый надзор привел к получению в среднем 54,3 отчета в месяц, тогда как традиционный надзор приводил к получению в среднем 6,8 отчета (P < 0,01). Анализ затрат показал, что затраты на краудсорсинговый надзор составили 72,4 доллара США из расчета на выявленный случай.

**Вывод:**

Предложенный метод с применением краудсорсинга превзошел традиционный надзор и является многообещающим с точки зрения улучшения надзора за забытыми тропическими болезнями. Ценность метода заключается в его способности выявлять вновь возникающие заболевания и поддерживать надзор после элиминации.

## Introduction

Neglected tropical diseases are a group of debilitating illnesses that disproportionately affect the world's poorest populations. These diseases are prevalent in marginalized communities with limited access to basic health care, clean water and sanitation. The global burden of neglected tropical diseases is immense, affecting an estimated 1.7 billion individuals, mainly in low- and middle-income countries.^1^^–^^3^ Despite the World Health Organization (WHO) neglected tropical diseases road map (2012–2020)^4^ and the United Nations sustainable development goals (specifically SDG3), considerable challenges persist in disease surveillance and intervention strategies. WHO’s latest road map (2021–2030)^5^ outlines strategies for the control, elimination and eradication of neglected tropical diseases, and highlights the importance of effective surveillance and targeted deployment of interventions. However, traditional surveillance methods remain time-consuming, resource-intensive, expensive, limited in scope and often fail to cover the entire population and accurately capture disease prevalence. Factors such as stigma and reluctance to seek medical care further contribute to gaps in reporting.^6^^,^^7^ Moreover, traditional surveillance is ineffective under conditions that prevent access to locations where diseases may be endemic, such as during pandemics or civil unrest.^8^

In Nigeria, which accounts for about 25% of neglected tropical disease prevalence in the WHO African Region, neglected tropical diseases impose considerable health and social burdens, particularly in marginalized communities.^9^ Currently there is no active neglected tropical disease surveillance in the country, with prevalence data typically generated through passive surveillance (when individuals present at health facilities with symptoms). This approach is inadequate for mapping hotspots and deploying targeted interventions; thus there is critical need for new surveillance methods to improve disease control.

In this study we introduce and evaluate a crowdsourced, image-based morbidity hotspot surveillance method for neglected tropical diseases (hereafter referred to as crowdsourced surveillance). This crowdsourcing method uses smartphone technology to help improve real-time disease tracking, overcome the limits of traditional methods, and speed up progress towards global elimination targets.^9^ Here we present findings from a pilot implementation of crowdsourced surveillance in Nigeria and compare incidence data with that obtained by traditional methods.

## Methods

### Study design

We evaluated the crowdsourced surveillance method using a quantitative research approach embedded in an implementation research framework. We employed a quasi-experimental design to compare the effectiveness of this new approach with traditional surveillance methods for neglected tropical diseases across the study sites.

### Participants

We conducted the study in six states in southern Nigeria. Three states were selected as implementation sites; the other three were control sites. Site selection was based on the recorded prevalence of neglected tropical diseases, the co-endemicity of the target diseases and the presence of partner nongovernmental organizations to support data collection and community mobilization. Implementation states were carefully matched with control states based on similarities in key population characteristics, including demographics, education, occupation, religion and sociocultural factors. Ondo, Enugu and Cross River (implementation states) were respectively matched with Ogun, Imo and Akwa Ibom (control states).

Community members (including individuals with skin or eye manifestations suspected to be due to neglected tropical diseases) from 45 communities in these states, within 15 local government areas, were subjects in the study. Inclusion criteria required participants to have resided in the selected communities for at least one year and to be capable of providing informed consent.

### Intervention description

Crowdsourced surveillance is a community-driven digital tool designed to improve early detection and reporting of skin- and eye-related neglected tropical diseases in underserved settings.^9^ Target diseases include Buruli ulcer, leprosy, lymphatic filariasis, onchocerciasis and trachoma. Individuals with suspect skin or eye manifestations submit anonymized photographs via digital communication platforms (WhatsApp, Meta Platforms Inc., Menlo Park, United States of America; or Telegram, Dubai, United Arab Emirates) to a designated surveillance line. Trained community focal points, so called community contact people, help with image capture and submission in communities that lack smartphones or internet. Each image includes timestamp and geolocation data. An expert panel reviews images and diagnosis is confirmed when at least three experts agree.

### Implementation strategy

We piloted the intervention stepwise in three states over the 2-year period, from November 2022 to October 2024. The implementation strategy is described as follows:

#### Community engagement

We selected communities as outlined earlier. To ensure community buy-in, we engaged health authorities, state and local neglected tropical disease programme officers, and traditional rulers early on.

#### Stakeholder engagement

Following ethical approval, neglected tropical disease officers from the state conducted advocacy visits to gain political and institutional support. This helped identify suitable communities for the pilot implementation.

#### Community sensitization

Sensitization began with entering communities and meeting local leaders. Town criers mobilized residents to attend town hall meetings where we introduced the approach. Communities also selected locations for project signposts that served as information hubs.

#### Community focal points

Communities nominated community focal points based on agreed criteria, including trustworthiness, availability, and possession of a smartphone capable of capturing good-quality images. Community-led selection ensured local ownership and sustainability. No phones were provided, but each focal point received data stipends and information, education and communication materials. Each community selected one focal point. Following selection, the research team’s monitoring and evaluation officer trained the selected individual to identify neglected tropical disease symptoms using WHO’s manual,^10^ collect ethical data, capture smartphone images and use WhatsApp or Telegram. Training lasted approximately 30 minutes and took place on-site in a suitable area within the community. Community members also received basic training on image capture and transmission during sensitization. We evaluated training effectiveness using a structured, 20-question multiple-choice questionnaire pre- and post-training, covering identification of disease symptoms, ethics, image capture and referral procedures. Knowledge levels were categorized as good (60–100%), average (30–59%) and poor (< 30%).

#### Image submission

Community members or focal points submitted images of individuals with a suspected neglected tropical disease via WhatsApp or Telegram. In areas with poor connectivity, images were stored on the focal point’s smartphones and uploaded later.

#### Image review

Submissions were reviewed by our research team and independently assessed by a panel of five clinical experts (including dermatologists and parasitologists). Disease was confirmed if three out of five experts concurred, based on WHO diagnostic criteria.^10^

#### Referral

Individuals confirmed to have a neglected tropical disease were referred to treatment facilities or other partners. For example, individuals with hydrocele (swelling of the scrotum due to fluid buildup), a symptom of lymphatic filariasis, were referred and surgically treated through partner support in Ondo.

#### Feedback

We gave feedback to those submitting images either directly or through community focal points. This feedback loop ensured authentic reporting and strengthened community trust in the system. Currently, there is no centralized system for tracking treatment adherence. However, anecdotal feedback was collected through community focal points during their follow-up visits.

### Implementation

The full implementation period spanned 2 years, with different states having different start/end times for implementation during this period. Initial advocacy and stakeholder engagement took 3 months, followed by 2 months of community sensitization and mobilization. Community focal point training and deployment occurred over a 1-month period, with data collection and image submission lasting 4 months. Expert review and feedback loops continued concurrently during the reporting phase, and post-report activities such as integration dialogue (discussions with government stakeholders on how the method could be integrated into existing disease elimination programmes) lasted an additional 2 months. In total, over 60 collaborators participated in the implementation, including community focal points, expert reviewers, local health officials and neglected tropical disease programme officers.

### Data collection

The crowdsourced surveillance approach, as outlined in its protocol,^9^ allowed community members to report suspected manifestations of neglected tropical diseases, accompanied by demographic and geographic metadata. We used location coordinates of individuals with confirmed disease to generate hotspot maps of neglected tropical diseases using geographic information system software ArcGIS (Environmental Systems Research Institute, Inc., Redlands, United States).

We used a standardized data extraction form to collect monthly data on individuals with a neglected tropical disease from surveillance records in both intervention and control locations during the study period. This enabled direct comparison of the two surveillance methods.

### Data analysis

We analysed demographic and geographic data using IBM SPSS Statistics (Armonk, New York, USA),^11^ and noted results in frequency distribution tables. We compared incidence data from the two surveillance methods using the Kruskal–Wallis non-parametric test, setting statistical significance at a *P*-value: < 0.05. Finally, we assessed inter-rater agreement among image reviewers using Fleiss’ Kappa statistics, which demonstrated substantial agreement (*κ* = 0.78).

### Ethics

The study received ethical approval (NHREC/01/01/2007–16/01/2023) from the National Health Research Ethics Committee of Nigeria. We informed participants about the project through advertisements, and considered the transmission of images and data as implied consent. Automated responses on WhatsApp or Telegram asked image senders to confirm consent. Community focal points obtained written informed consent for participants without smartphones, with parents or caregivers providing consent for children and other vulnerable groups. We collected hard copies of these signed consent forms and stored them in a secure steel cabinet. We stored electronic consent in a password-protected and encrypted electronic database.

## Results

### Training effectiveness

Our assessment of the training effectiveness of community focal points revealed a large improvement in knowledge following training. Before training, 80% (36/45) of participants showed poor knowledge and none showed good knowledge. After training, no one showed poor knowledge and 84.4% (38/45) of participants showed good knowledge, an increase of 84.4%. The proportion of participants in the average category decreased from 20% (9/45) to 15.6% (7/45; Table 1).

**Table 1:** Assessment of training effectiveness of community focal points in the evaluation of crowdsourced surveillance for neglected tropical diseases, Nigeria, March – November 2023

Knowledge level	No. (%)^a^		% change
	Pre-training	Post-training	
Good	0 (0.0)	38 (84.4)	84.4
Average	9 (20.0)	7 (15.6)	−4.4
Poor	36 (80.0)	0 (0)	−80.0
**Mean score, % (SD)**	**29.0 (12.0)**	**78.8 (11.2)**	**–**

SD: standard deviation.

^a^ Numbers and percentages are presented if not otherwise indicated.

Note: we categorized the knowledge level using the scores from a 20-question multiple-choice questionnaire, where a score of 60–100% was categorized good level, 30–59% as average and 30% as poor.

### Overview of findings

Table 2 presents a summary of the data from the crowdsourced surveillance. A total of 512 images were submitted, with community focal points facilitating over 80% (437/512) of reports. Expert reviewers confirmed 75.4% (386/512) of the submissions as showing individuals with a neglected tropical disease.

**Table 2:** Summary of crowdsourced data in the evaluation of crowdsourced surveillance for neglected tropical diseases, Nigeria, March – November 2023

State	No.			No. (%)			
	Communities sampled	Total population of sampled communities		Total crowdsourced images received	Source		Confirmed as a neglected tropical disease
					Community focal point	Community member	
Ondo	15	185 099		217 (42)	192 (88)	25 (12)	175 (81)
Enugu	15	140 556		153 (30)	126 (82)	27 (18)	105 (69)
Cross River	15	151 483		142 (28)	119 (84)	23 (16)	106 (75)
**Total**	**45**	**477 138**		**512 (100)**	**437 (85)**	**75 (15)**	**386 (75)**

### User demographics

Demographic characteristics of crowdsourced surveillance users by sex, age, marital status, occupation, manifestation duration, receipt of intervention and intervention type were tabulated (see online repository).^12^ Of the users, 54.3% (278/512) were male. The 55–65 years age group accounted for 22.5% (115/512) of users and the mean age was 53 years (standard deviation: 20.7). The majority were farmers (56.4%; 289/512). Notably, 42.6% (218/512) of users had lived with their condition for 1–5 years. Additionally, 85.3% (437/512) of users reported receiving no prior intervention.

### Surveillance method comparison

Table 3 compares crowdsourced surveillance in the three implementation states with traditional surveillance for neglected tropical diseases over a period of four months; note that different states had different start and end dates. In Ondo, crowdsourced surveillance garnered a total of 217 reports in four months, averaging 54.3 reports per month. This compared to averages of only 6.8 reports per month using traditional surveillance, and 1.5 reports in Ogun (Ondo’s control state). In Enugu, crowdsourced surveillance generated a total of 153 reports, while the traditional method and control state (Imo) yielded none. Similarly, in Cross River, crowdsourced surveillance generated a total of 142 reports, while the traditional method and control state (Akwa Ibom) yielded none. The mean difference between crowdsourced surveillance data and traditional surveillance data was statistically significant (*P* < 0.01).

**Table 3:** Comparison of data generated using crowdsourced versus traditional surveillance in the evaluation of crowdsourced surveillance for neglected tropical diseases, Nigeria, March – November 2023

Month	Surveillance type, no.														*P*
	Ondo			Ogun (control for Ondo)		Enugu			Imo (control for Enugu)		Cross River			Akwa Ibom (control for Cross River)	
	Crowd-sourced	Traditional		Traditional		Crowd-sourced	Traditional		Traditional		Crowd-sourced	Traditional		Traditional	
1	62	8		3		50	0		0		36	0		0	0.0086
2	49	6		3		30	0		0		39	0		0	
3	50	3		0		36	0		0		32	0		0	
4	56	8		0		37	0		0		35	0		0	
**Total (mean)**	**217 (54.3)**	**25 (6.8)**		**6 (1.5)**		**153 (38.3)**	**0 (0)**		**0 (0)**		**142 (35.5)**	**0 (0)**		**0 (0)**	

Note: we used the Kruskal–Wallis test to calculate significance.

### Types of diseases

Table 4 depicts the type of neglected tropical disease reported and confirmed using crowdsourced surveillance. Onchocerciasis was reported in 187 individuals, lymphatic filariasis in 102 individuals and trachoma in 40 individuals across the three states.

**Table 4:** Frequency distribution of various neglected tropical diseases using crowd surveillance in the evaluation of its use for neglected tropical diseases, Nigeria, March – November 2023

Neglected tropical disease	No. of individuals confirmed to have the disease			
	Ondo	Enugu	Cross River	Total
Buruli ulcer	17	12	9	38
Helminthiasis	1	0	0	1
Leprosy	7	1	7	15
Lymphatic filariasis	56	26	20	102
Onchocerciasis	73	45	69	187
Scabies	1	0	0	1
Schistosomiasis	2	0	0	2
Trachoma	18	21	1	40
**Total**	**175**	**105**	**106**	**386**

**Fig. 1:**
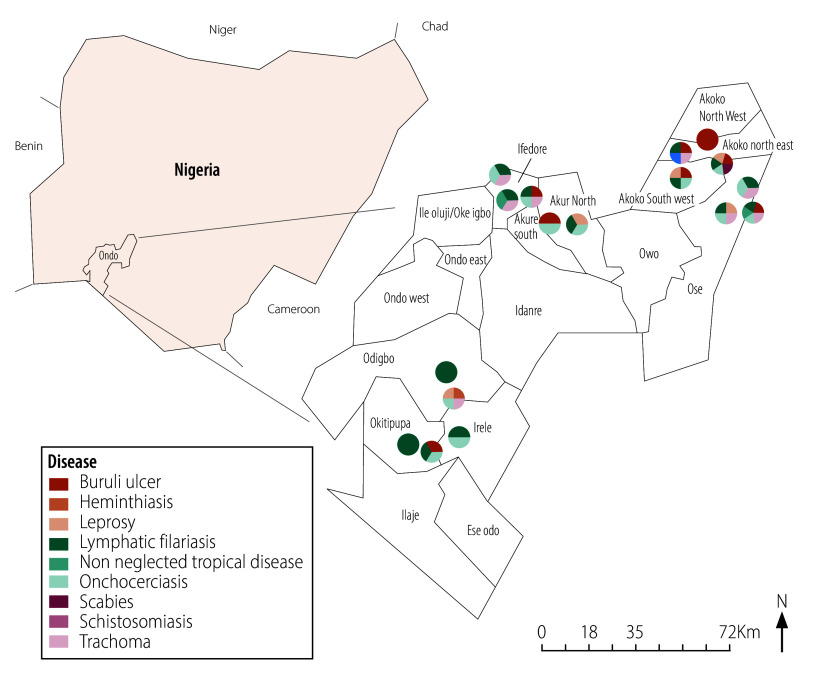
Morbidity hotspot maps for Ondo state, generated by crowdsourced surveillance in the evaluation of crowdsourced surveillance for neglected tropical diseases, Nigeria, March – November 2023

**Fig. 2:**
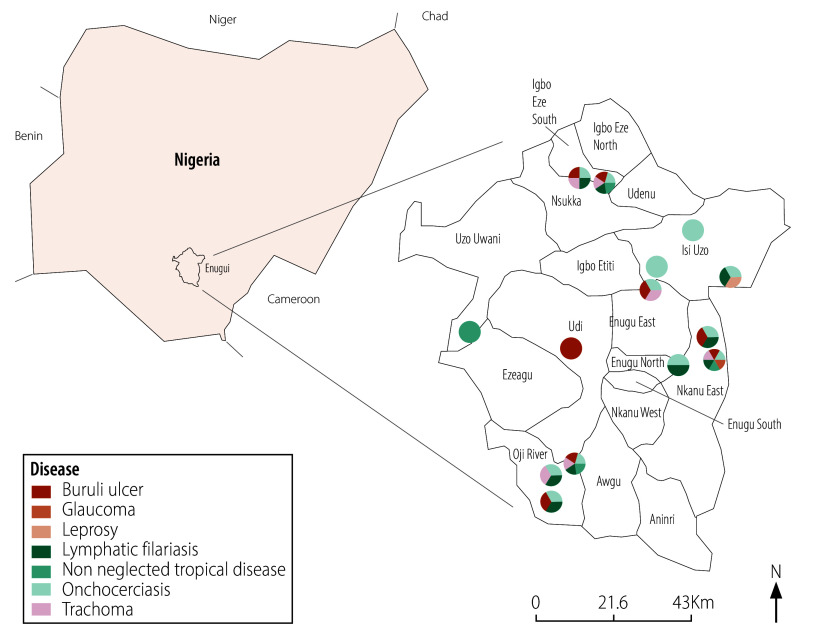
Morbidity hotspot maps for Enugu state, generated by crowdsourced surveillance in the evaluation of crowdsourced surveillance for neglected tropical diseases, Nigeria, March – November 2023

**Fig. 3:**
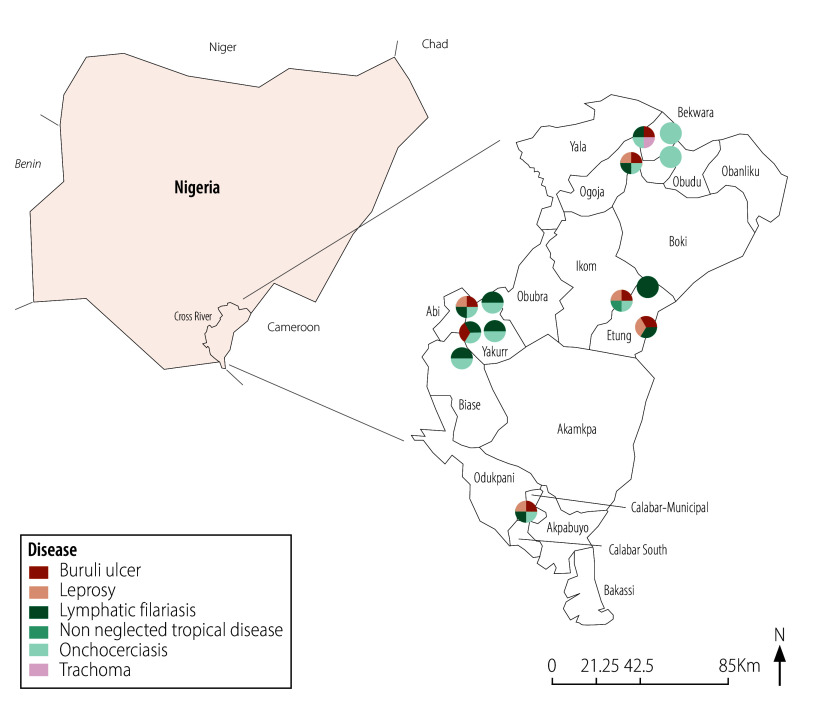
Morbidity hotspot maps for Cross River state, generated by crowdsourced surveillance in the evaluation of crowdsourced surveillance for neglected tropical diseases, Nigeria, March – November 2023

Fig. 1, Fig. 2 and Fig. 3 illustrates neglected tropical disease morbidity hotspot maps for the three states, indicating locations where neglected tropical diseases were identified using crowdsourced surveillance.

### Surveillance potential

**Fig. 4:**
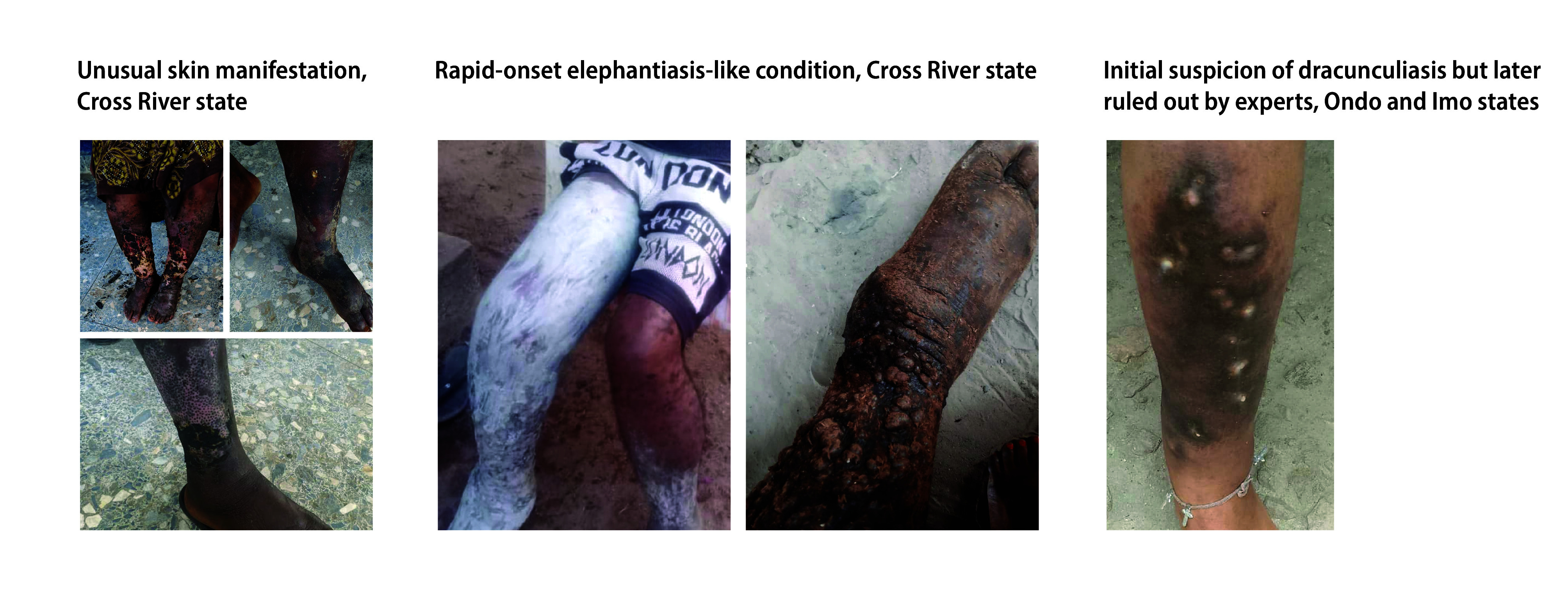
Images of unusual manifestations obtained during the evaluation of crowdsourced surveillance for neglected tropical diseases, Nigeria, March – November 2023

Fig. 4 shows unusual manifestations that were reported through the crowdsourced surveillance method. In Cross River, there were reports of blistering, burn-like symptoms on swollen limbs despite affected individuals having no known exposure to burns or chemicals. A 37-year-old man in Cross River also reported rapid-onset, severe elephantiasis-like symptoms, unusual given the typically slow progression of the condition. Two individuals initially suspected of having dracunculiasis were also reported: a 19-year-old in Ondo had described a thread-like worm emerging from her leg; and a child in Imo described similar symptoms. Although expert assessment ruled out dracunculiasis, the symptoms in these two demonstrate the crowdsourced surveillance method’s potential for identifying emerging conditions and facilitating post-elimination surveillance with the continuous trickling-in of photo and metadata where the project was piloted.

### Cost

Of the 512 individuals reporting a suspected disease, reviewers confirmed that 386 (75.4%) individuals had a neglected tropical disease. The total direct cost incurred for implementing the approach, considering only the cost of expert analysis (9000 United States dollars, US$) and information, education and communication materials (US$ 28 968), was US$ 37 968. The cost per identified individual with any suspected disease was US$ 74.2 (US$ 37 968/512 cases), while the cost per person with a confirmed disease was US$ 98.4 (US$ 37 968/386 cases).

## Discussion

Our project demonstrates the effectiveness of crowdsourced surveillance in enhancing neglected tropical disease detection and contributing to global elimination.^5^ Particularly valuable in resource-constrained settings, crowdsourced surveillance’s success in this study aligns with previous successes in crowdsourced and image-based disease surveillance, including diabetic retinopathy screening^13^ and foodborne illness surveillance.^14^

Over the 4-month period of data collection the project received more than 500 photographs across three states, with three quarters confirmed as neglected tropical diseases. Crowdsourced surveillance generated more reports per month than traditional methods, highlighting its ability to engage communities and improve detection of individuals with disease. Similar approaches have shown improved disease detection and reporting rates in other community-based surveillance studies.^15^

In our pilot, older adults submitted the most reports, while younger individuals submitted least. This finding suggests a need for targeted awareness campaigns to encourage younger populations to participate in the process. A higher proportion of older adults reporting may reflect the long latency of these diseases or delayed recognition of problems. Stigma may also contribute to late reporting, an issue that crowdsourced surveillance addresses as it enables private reporting. Strengthening community engagement and education, central to crowdsourced surveillance, could further reduce delays in seeking care.

More women compared to men used crowdsourced surveillance, suggesting gender differences in reporting. This finding may suggest a greater burden of disease among women or more proactive health-seeking behaviour. Further research is needed to explore this. Gender disparities have been documented elsewhere, for example in Uganda, where women were more likely to have schistosomiasis due to greater exposure to water sources.^16^ Most users were farmers, reflecting the rural, agrarian nature of most-affected communities. These demographic insights are essential for designing targeted interventions.

Notably, 85% of subjects were not receiving intervention for their conditions, pointing to substantial gaps in health-care delivery and probably the health-seeking behaviour of the people. This finding underscores the critical role of innovative surveillance methods in narrowing the gap between detection of individuals with disease and treatment. Similar gaps have been observed in other countries such as Brazil and India, often driven by limited health infrastructure, stigma and low awareness.^17^^,^^18^

Comparing crowdsourced surveillance with traditional approaches reveals its superior effectiveness. Higher reporting rates and quicker data collection because subjects just need to send pictures without having to leave their houses, highlight the potential of crowdsourcing to strengthen surveillance. Traditional methods, often labour-intensive and limited in reach, tend to miss individuals with disease symptoms from remote areas. Crowdsourced surveillance addresses these challenges through mobile technology and community involvement, consistent with research on mobile health interventions in other contexts.^19^^,^^20^

The fact that crowdsourced surveillance has potential to indicate emerging and re-emerging health conditions, and atypical manifestations of known conditions, suggests its utility beyond neglected tropical disease surveillance. The suspected dracunculiasis incidence, although ruled out, also exemplifies potential for effective post-elimination surveillance.

We observed a detection cost of approximately US$ 74 for all reports and US$ 98 for individuals confirmed to have a neglected tropical disease, comparing favourably with traditional surveillance costs and mobile health interventions (US$ 100–150).^21^^,^^22^ Crowdsourced surveillance significantly improved disease detection and community engagement. Aligned with WHO’s call for scalable, resource-conscious innovations,^23^ the model leverages existing community health structures by training frontline workers such as community health extension workers and environmental health officers, reducing the need for costly new infrastructure and reinforcing primary health care. Scaling up and conducting operational research is essential to assess the model’s long-term cost benefits and adaptability across other diseases and geographical contexts.

Sustaining crowdsourced surveillance requires funding, capacity-building, logistical support and investment in training, infrastructure and monitoring. Recommendations include regional scale-up, enhanced community mobilization and local leadership involvement. Future expansion could target other diseases with visible symptoms, such as other skin diseases, malaria or emerging zoonoses, and other regions.

Crowdsourced surveillance could be integrated into Nigeria’s national neglected tropical disease surveillance system by training health workers already present in primary health care facilities to identify symptoms, capture images ethically and report data as part of their routine duties. Trained health workers could be equipped with the WHO Skin NTDs app (WHO, Geneva, Switzerland), enabling them to confirm disease themselves and eliminating the need for an expert panel. This approach would embed surveillance in the health system, cut costs, enhance sustainability and strengthen links between detection and treatment services.

Despite its success, implementing crowdsourced surveillance faced challenges. Reliance on smartphones and stable internet affected reporting in remote communities with poor digital access. Although community focal points helped bridge this gap, coverage likely remained uneven. Logistical issues, such as upload delays and the need for continuous monitoring and technical support, posed further hurdles. In urban areas, weaker social cohesion made it harder to identify reliable community focal points. These limitations underscore the need for strengthened infrastructure, digital literacy and government commitment to scale up and sustain the intervention. Sampling bias may have arisen from unequal smartphone access and reliance on community focal points. Reporting bias could arise from a focus on visible or severe symptoms. These limitations may affect the generalizability of prevalence data.

In conclusion, crowdsourced surveillance represents a leap forward in surveillance of neglected tropical diseases by enabling real-time, community-led reporting. This method improves detection, enhances privacy and reduces stigma. Investment in training, infrastructure and logistics is essential for scale-up. Targeted awareness campaigns, especially among youth, should increase participation. Sustained funding and long-term feasibility studies will help evaluate integration into routine surveillance and ensure its effectiveness, sustainability and scalability for broader disease control.
